# Effects of clonidine and scopolamine on multiple target detection in rapid serial visual presentation

**DOI:** 10.1007/s00213-015-4111-y

**Published:** 2015-10-28

**Authors:** Stephen B. R. E. Brown, Heleen A. Slagter, Martijn S. van Noorden, Erik J. Giltay, Nic J. A. van der Wee, Sander Nieuwenhuis

**Affiliations:** Cognitive Psychology Unit, Institute of Psychology, Leiden University, Leiden, The Netherlands; Leiden Institute for Brain and Cognition, Leiden, The Netherlands; Brain and Cognition Unit, Department of Psychology, University of Amsterdam, Amsterdam, The Netherlands; Department of Psychiatry, Leiden University Medical Center, Leiden, The Netherlands; Amsterdam Brain and Cognition Center, University of Amsterdam, Amsterdam, The Netherlands

**Keywords:** Attentional blink, Rapid serial visual presentation, Temporal attention, Noradrenaline, Acetylcholine

## Abstract

**Rationale:**

The specific role of neuromodulator systems in regulating rapid fluctuations of attention is still poorly understood.

**Objectives:**

In this study, we examined the effects of clonidine and scopolamine on multiple target detection in a rapid serial visual presentation task to assess the role of the central noradrenergic and cholinergic systems in temporal attention.

**Method:**

Eighteen healthy volunteers took part in a crossover double-dummy study in which they received clonidine (150/175 μg), scopolamine (1.2 mg), and placebo by mouth in counterbalanced order. A dual-target attentional blink task was administered at 120 min after scopolamine intake and 180 min after clonidine intake. The electroencephalogram was measured during task performance.

**Results:**

Clonidine and scopolamine both impaired detection of the first target (T1). For clonidine, this impairment was accompanied by decreased amplitudes of the P2 and P3 components of the event-related potential. The drugs did not impair second-target (T2) detection, except if T2 was presented immediately after T1. The attentional blink for T2 was not affected, in line with a previous study that found no effect of clonidine on the attentional blink.

**Conclusions:**

These and other results suggest that clonidine and scopolamine may impair temporal attention through a decrease in tonic alertness and that this decrease in alertness can be temporarily compensated by a phasic alerting response to a salient stimulus. The comparable behavioral effects of clonidine and scopolamine are consistent with animal studies indicating close interactions between the noradrenergic and cholinergic neuromodulator systems.

## Introduction

Temporal attention—the dynamic changes in attention on a fast timescale—is widely studied because it is crucial for organisms to be able to prioritize and accurately identify incoming information, for example, to make successful decisions. In recent years, the neuromodulatory basis of temporal attention has attracted considerable scientific interest. One theory relates temporal attention to the noradrenergic neuromodulator system. This theory is founded on the idea that the release of noradrenaline by the locus coeruleus (LC) adjusts the gain of post-synaptic neurons, thereby modulating these neurons’ responsivity (Servan-Schreiber et al. (1990). The LC has been demonstrated to fire phasically following the presentation of task-relevant or salient stimuli (Aston-Jones et al. 2000), and these phasic bursts are temporally closely related to behavioral responses (Bouret and Sara 2004). These findings suggest that the phasic LC response acts as a temporal attention filter that selectively facilitates the processing of motivationally significantly stimuli (Aston-Jones and Cohen 2005; Nieuwenhuis et al. 2005a).

Rapid changes in temporal attention are commonly studied with the attentional blink task, in which participants have to identify two targets that are embedded in a rapid serial visual stream (RSVP) of distractor stimuli. Participants are usually able to accurately identify the first of those targets (T1). The crucial finding in this task is that when the second target (T2) follows the first target within 200–400 ms, participants are often unable to report T2 accurately (Raymond et al. 1992; Chun and Potter 1995). This phenomenon is referred to as the attentional blink.

Nieuwenhuis and colleagues have proposed a theory in which the attentional blink reflects the temporal dynamics of the noradrenergic system (Nieuwenhuis et al. 2005b; see also Warren et al. 2009). This theory assumes that identification of T1 is associated with a transient burst of arousal and concomitant phasic firing of the LC. Following this T1-related phasic burst, LC neurons enter a refractory period of reduced firing. During this period, no noradrenaline-mediated facilitation of stimulus processing can occur. The characteristic attentional blink window of 200–400 ms corresponds to the duration of this refractory period, which would explain why participants often fail to detect T2 if it is presented within this time window. This theory also accounts for the phenomenon of lag-1 sparing (Raymond et al. 1992; Hommel and Akyürek 2005): Participants generally do detect T2 if it directly follows T1 (i.e., at “lag 1”). When there is such a close temporal proximity of T1 and T2, detection of T2 is proposed to benefit from the phasic noradrenaline burst elicited by T1 (Usher et al. 1999).

There is direct empirical evidence for the involvement of the noradrenergic system in target detection under rapid serial visual presentation conditions. For example, antagonization of noradrenergic β receptors by propranolol led to impaired T2 identification in humans (de Martino et al. 2008). Furthermore, patients with dopamine-β-hydroxylase deficiency, a rare genetic syndrome characterized by the complete absence of noradrenaline, were shown to have a larger attentional blink than healthy controls, and this impairment was restored by treatment with a synthetic precursor of noradrenaline (Jepma et al. 2011). However, when Nieuwenhuis et al. (2007) used the noradrenergic α_2_ agonist clonidine to attenuate noradrenergic baseline activity, they did not find a reliable decrease in T2 identification accuracy, a finding that seems at odds with the theory of Nieuwenhuis et al. (2005b).

The goal of the present experiment was to replicate the study by Nieuwenhuis et al. (2007) but with two improvements: a crossover design instead of a between-subject design to increase statistical power and an increased dose of clonidine to induce a more pronounced attenuation of the noradrenergic system. Furthermore, we recorded the electroencephalogram (EEG) to acquire more insight into the electrophysiological correlates of treatment effects on RSVP performance.

We tested 18 healthy adult participants in a double-blind placebo-controlled randomized crossover design. Participants received, in different test sessions, a single dose of clonidine, scopolamine, and placebo. Clonidine is a centrally acting α_2_ agonist that attenuates baseline noradrenergic activity by agonizing pre-synaptic α_2_ receptors and decreases the amplitude of the human P3 component, a putative electrophysiological correlate of phasic noradrenaline release (Nieuwenhuis et al. 2005a; Pineda et al. 1989). Due to its antihypertensive properties, the main indication of this drug is hypertension, but it is also indicated to reduce menopausal hot flashes and as an adjuvant in opiate withdrawal treatment. Its most common side effects are dizziness, sedation, orthostatic hypotension, and dry mouth. If the attentional blink is mediated by a phasic noradrenergic burst following presentation of T1 and clonidine decreases this phasic burst, then clonidine may be expected to reduce the attentional blink. Notably, previous event-related potential (ERP) studies have associated the attentional blink with a larger or a delayed T1-elicited P3 (Martens et al. 2006; Sergent et al. 2005; Slagter et al. 2007). We were thus specifically interested in possible effects of clonidine on the T1-elicited P3.

The involvement of the cholinergic system in temporal attention has been investigated less extensively than that of noradrenaline. Previous empirical work has focused on the nicotinic cholinergic system and has not studied RSVP performance but other temporal attention tasks like temporal cuing (e.g., Beane and Marrocco 2004; Stewart et al. 2001). Therefore, we also administered the muscarinic antagonist scopolamine, a drug with a sedation profile comparable to that of clonidine. Scopolamine has pronounced antiemetic properties, and its main indications are therefore (post-operative) nausea and motion sickness, but it is also indicated to treat gastrointestinal spasms. Its main side effects are dry mouth, bradycardia, and mydriasis. The use of scopolamine allowed us to gain insight into the role of the cholinergic muscarinic system in temporal attention and to test whether any treatment effects are specific to the noradrenergic system.

## Methods and materials

### Participants

Eighteen healthy young adults (15 women), aged 18–26 years (mean age 21 years), drafted through Leiden University’s participant recruitment system, took part in three 4.5-h experimental sessions in return for €140. Only participants with a systolic blood pressure above 100 mmHg, a diastolic blood pressure above 70 mmHg, and a heart frequency over 65 beats per minute in rest were included in the study (cf. Nieuwenhuis et al. 2007). All participants underwent a medical screening which included a routine physical examination prior to being included in the experiment: Only healthy individuals without a history of neurological or psychiatric disorders were allowed to participate. Participants took no prescribed medication and did not smoke; participants were instructed to abstain from using recreational drugs, caffeine, or alcohol 15 h prior to the study. Female participants were asked whether they were pregnant or thought they might be pregnant to preclude pregnant females from participating. Participants received a single oral dose of clonidine, a single oral dose of scopolamine (1.2 mg), and a placebo in a randomized, double-blind, counterbalanced double-dummy crossover design. The first 11 participants received a clonidine dose of 175 μg. As the 11th participant showed an unexpected large drop in blood pressure of 35 mmHg systolic, but without clinical consequences, 60 min after the ingestion of clonidine 175 μg (blind was broken by the supervising physician), we decided to reduce the dose of clonidine to 150 μg for the final seven participants. Preliminary repeated measures ANOVAs with dose as between-subject factor revealed no reliable main effect of dose or interactions including this factor, so in the analyses reported below, the 18 participants are pooled. Clonidine, scopolamine, and placebo were administered during three separate test sessions, spaced 1 week apart. The study was approved by the medical ethics committee of the Leiden University Medical Center. Written informed consent was obtained from all participants prior to inclusion in the study.

### Task

Participants performed an attentional blink task. Each trial started with a 500-ms fixation point (black plus sign on light gray background, visual angle 0.6° × 0.6°), followed by a 2-s blank, after which a RSVP stream of 22 uppercase letters was presented centrally (visual angle of each letter approximately 0.7° × 0.7°) on an IIyama Vision Master CRT monitor with a refresh rate of 100 Hz, using E-Prime 2.0 (Psychology Software Tools, Sharpsburg, PA). Each letter was randomly drawn without replacement from the alphabet and presented for 70 ms, followed by a blank of 30 ms. The letters *I*, *O*, *Q*, and *S* were left out, as they resemble digits too much. On each trial, two letters were replaced by digits (range 2–9, chosen randomly without replacement): targets 1 and 2 (T1 and T2). T2 was presented three to six temporal positions from the end of the stream. The temporal distance between T1 and T2 was either one (12.5 % of trials), two (37.5 % of trials), three (37.5 % of trials), or seven items (12.5 % of trials), corresponding to lags of 100, 200, 300, and 700 ms. Immediately after the end of the RSVP stream, participants were asked to identify T1 and T2 by typing them, in order, on a standard keyboard. The task consisted of six blocks of 40 trials each and was preceded by a practice block of 12 trials, in which feedback on the participants’ performance was given on every trial (e.g., a display of “+ −” indicated that a participant had entered T1 correctly and T2 incorrectly).

### Procedure

Each participant was tested at approximately the same time of day. During every test session, participants received a capsule of clonidine or placebo at 09.35 AM and a capsule of scopolamine or placebo at 10.35 AM. The different kinetic profiles of clonidine and scopolamine necessitated administrations at different times prior to testing. This double-dummy design resulted in one clonidine session (i.e., clonidine verum plus scopolamine placebo), one scopolamine session (clonidine placebo plus scopolamine verum), and one placebo session (clonidine plus scopolamine placebos). To eliminate any possible confound of drug order, we stratified this factor by distributing the six possible drug orders evenly across participants.

**Fig. 1:**
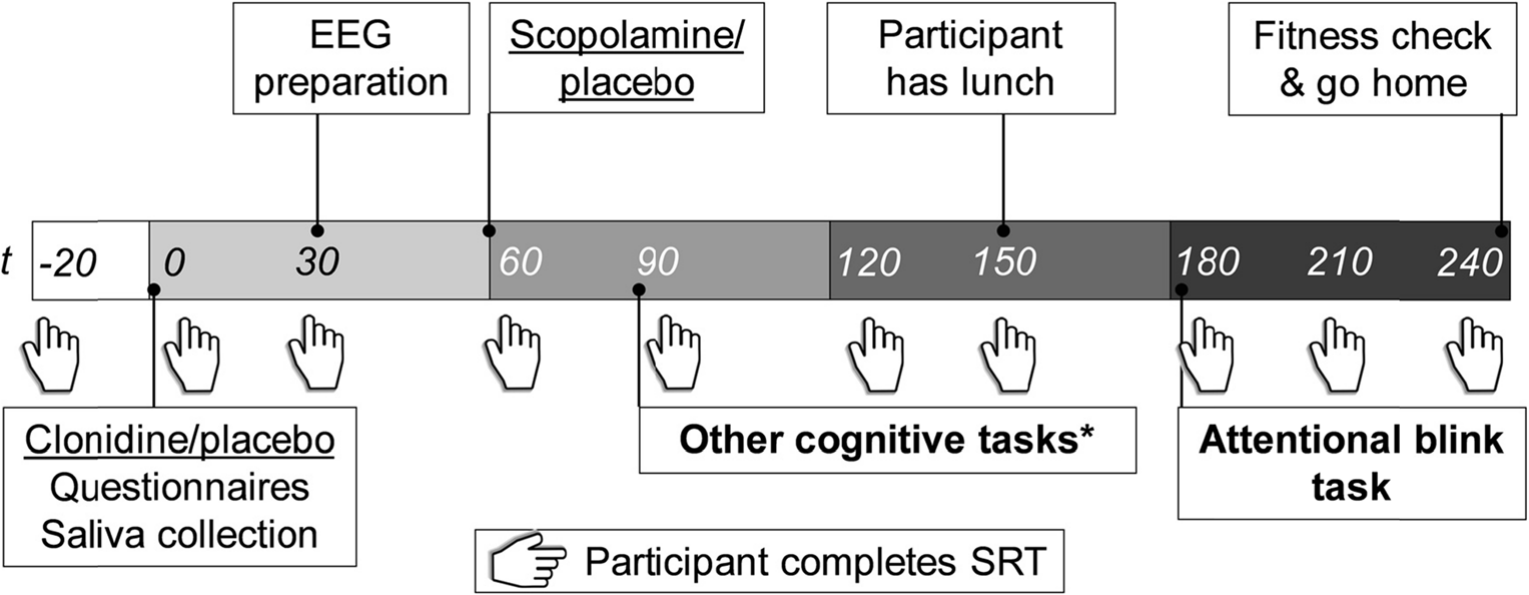
Timeline of the procedure in each test session. *Connector lines* indicate the start of an event. Each cognitive task lasted approximately 30 min. Participants’ blood pressure and heart rate were measured at baseline (*t* = −20) and then every 15 min, starting at *t* = 0. Results from the other cognitive tasks are reported elsewhere (*asterisk*) (Brown et al. 2015a, b). *SRT* simple reaction time task

The procedure in each test session is illustrated in Fig. 1. At the start of each session (*t* = −20 min), a peripheral intravenous cannula was placed and connected to an intravenous 0.9 % NaCl (saline) drip to be able to increase blood pressure through volume expansion and to have an entryway to administer escape medication in the case of a severe drop in tension and/or heart frequency. Furthermore, three cardio electrodes were applied to the participant’s chest and connected to an electrocardiography (ECG) monitor. To measure the sedative, alertness-reducing properties of clonidine and scopolamine, we administered a 40-trial simple reaction time task upon a participant’s arrival in the lab, as well as right before and after the participant performed the attentional blink task. Participants had to respond as quickly as possible whenever a white circle appeared on the computer screen. Stimulus onset asynchrony was jittered between 500 and 1250 ms, with a mean of 1000 ms; this task lasted less than 2 min.

At *t* = 0 min, participants ingested a microcrystalline cellulose-filled capsule with either clonidine or placebo. Clonidine has well-established antihypertensive properties: Therefore, blood pressure and heart rate were monitored four times an hour from *t* = 0 onwards for participant safety with an Omron M10-IT automatic sphygmomanometer. At *t* = 60 min, participants ingested a microcrystalline cellulose-filled capsule with either scopolamine or placebo.

At *t* = 180, participants performed the attentional blink task which lasted approximately 30 min; during the 90 min prior to this time point, participants performed three unrelated cognitive tasks (Brown et al. 2015a, b). Participant fitness was checked at *t* = 240, and participants were sent home via public transportation if their blood pressure and heart rate were close to the values measured at *t* = −20; if their blood pressure and heart rate had not returned to normal yet, they were kept for further observation. At the end of the third test session, participants received their financial compensation.

### EEG recording and analyses

We recorded EEG from 64 Ag/AgCl scalp electrodes and from the left and right mastoids. We measured the horizontal and vertical electro-oculogram (EOG) using bipolar recordings from electrodes placed approximately 1 cm lateral of the outer canthi of the two eyes and from electrodes placed approximately 1 cm above and below the participant’s right eye. The EEG signal was pre-amplified at the electrode to improve the signal-to-noise ratio and amplified with a gain of 16× by a BioSemi ActiveTwo system (BioSemi B.V., Amsterdam). The data were digitized at 24-bit resolution with a sampling rate of 512 Hz using a low-pass fifth-order sinc filter with a half-power cutoff of 102.4 Hz. Each active electrode was measured online with respect to a common mode sense (CMS) active electrode producing a monopolar (non-differential) channel and was referenced offline to the average of the left and right mastoids. Data were high-pass filtered at 0.1 Hz and low-pass filtered at 30 Hz. Ocular and eyeblink artifacts were corrected using the method of Gratton et al. (1983). Epochs with other artifacts (a gradient greater than 30 μV, slow drifts [>300 μV/200 ms], and low activity [<0.50 μV/100 ms]) were discarded (placebo 1.2 %, clonidine 1.3 %, and scopolamine 2.5 %). Data were epoched from −100 to 600 ms relative to the onset of T1 and then averaged. A baseline, computed as the average signal activity across the 100 ms prior to T1, was subtracted for each ERP. Data pre-processing was performed in Brain Vision Analyzer 2 (Brain Products, Gilching, Germany).

As the active compounds only reliably influenced T1 accuracy (regardless of lag) and given previous studies linking the T1-evoked P3 to the attentional blink (e.g., Martens et al. 2006; Sergent et al. 2005; Slagter et al. 2007), we focused our electrophysiological analyses on T1-evoked potentials. Using matlab (The MathWorks, Natick, MA), we analyzed the ERP elicited by T1 with a sliding-window approach to examine whether clonidine and scopolamine differed from placebo, focusing in particular on electrodes Cz, CPz, and Pz, where the P3 was largest in amplitude. We collapsed T1-locked ERPs across lags, split the ERPs for each treatment and each participant into 19.5-ms windows, starting at *t* = 0 (i.e., 0–19.5, 21.5–39 ms, etc.), and then, for each window separately, submitted the average amplitudes to paired sample *t* tests with treatment (clonidine or scopolamine vs. placebo) as independent variable.

## Results

Greenhouse-Geisser corrections were applied whenever the assumption of sphericity was violated; in such cases, uncorrected degrees of freedom are reported.

### Physiological and alertness data

**Fig. 2:**
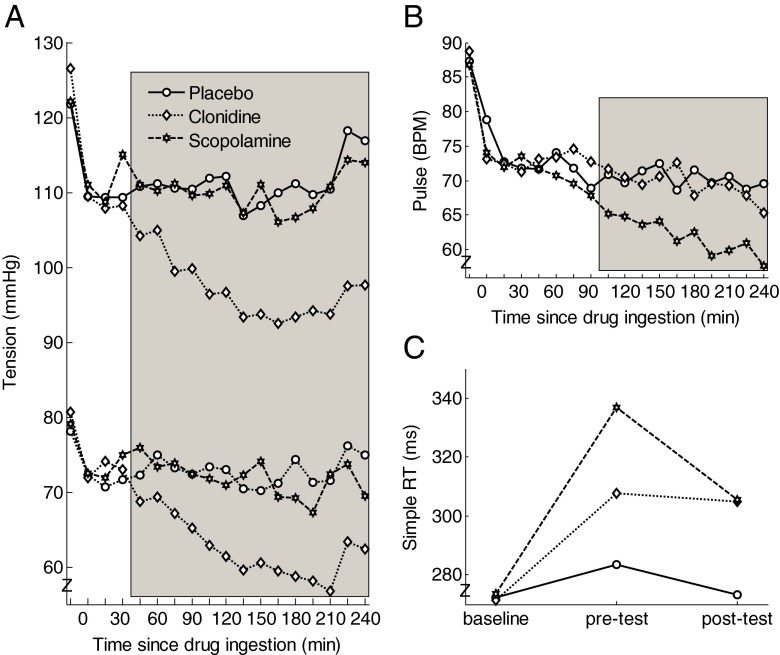
**a** Blood pressure data for the three treatments. The *shaded gray area* indicates significant pairwise comparisons between clonidine and placebo (*p* < .05). **b** Heart frequency for the three treatments. The *shaded gray area* indicates significant pairwise comparisons between scopolamine and placebo (*p* < .05). **c** Results from a simple reaction time task, administered at the start of the test session (baseline) and right before (pre-test) and after (post-test) participants performed the attentional blink task

Figure 2a shows that, as expected, clonidine lowered systolic (mean tension 101 mmHg) and diastolic (65 mmHg) blood pressure relative to placebo (mean tension 112/73 mmHg), also during performance of the attentional blink task (*t* = 180–210), all *t*_17_ > 4.5, *p*s < .0005. The difference in systolic and diastolic blood pressure between placebo and scopolamine was not significant. Figure 2b shows that scopolamine (61/min), as expected, lowered heart frequency relative to placebo (71/min) and clonidine (69/min), also during performance of the attentional blink task, all *t*_17_ > 3.3, *p*s < .004.

To test the subjects’ alertness, we administered a simple reaction time task at baseline (arrival of participant), right before, and right after performing the attentional blink task. As expected, the baseline measurements did not differ between treatments, *F*(2, 34) < 1, *p* = .95. To examine the effect of treatment on pre- and post-test measurements (Fig. 2c), we subtracted the baseline values from each of these measurements and submitted the difference scores to a 3 (treatment) × 2 (time point) repeated measures ANOVA. This analysis yielded a main effect of treatment, *F*(2, 34) = 4.8, *p* = .019, partial *η*^2^ = .22, but no interaction between treatment and time point, *F*(2, 34) = 1.6, *p* = .22. Pairwise comparisons indicated that clonidine and scopolamine reliably slowed down simple reaction time compared to placebo during both the pre-test (*t*_17_ = 2.1, *p* = .05 and *t*_17_ = 2.3, *p* = .04, respectively) and the post-test (*t*_17_ = 3.1, *p* = .006 and *t*_17_ = 3.3, *p* = .005, respectively). The differences between clonidine and scopolamine were not significant.

### Behavioral data

Trials on which T1 and T2 were accurately identified but in the wrong order were treated as correct (cf. Nieuwenhuis et al. 2007). Thus, the probability of guessing T1 and T2 correctly was 3.6 %.

**Fig. 3:**
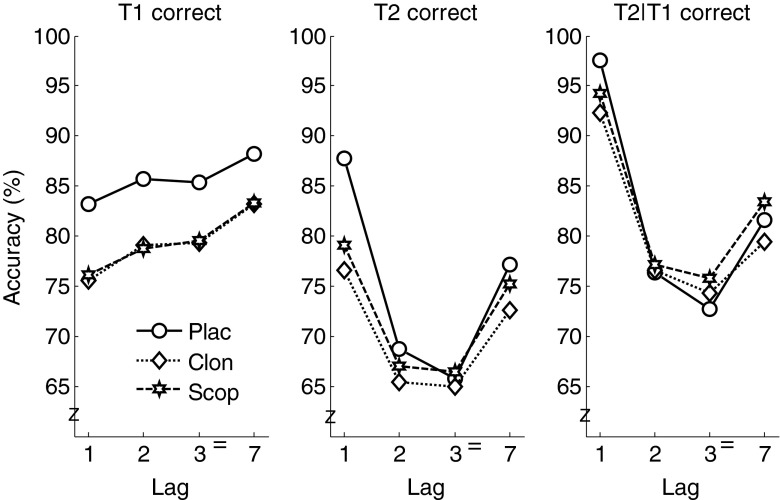
T1 identification accuracy (*left panel*), T2 identification accuracy (*middle panel*), and T2 identification accuracy (conditional upon T1 correct) as a function of treatment and lag

Figure 3 (left panel) shows average T1 accuracy as a function of treatment and lag. The main effect of treatment was significant, *F*(2, 34) = 4.4, *p* = .02, partial *η*^2^ = .21. Both clonidine (79.3 %, *t*_17_ = 2.5, *p* = .02) and scopolamine (79.4 %, *t*_17_ = 2.4, *p* = .03) decreased T1 identification accuracy relative to placebo (85.6 %). T1 identification accuracy increased with lag, *F*(3, 51) = 6.6, *p* = .001, partial *η*^2^ = .28. Treatment and lag did not interact, *F*(6, 102) = 0.2, *p* = .96.

To determine if the two drugs also decreased T2 accuracy, we examined average T2 accuracy, non-contingent on T1 identification, as a function of treatment and lag (Fig. 3, middle panel). Treatment did not reliably influence T2 identification accuracy, *F*(2, 34) = 1.6, *p* = .21, partial *η*^2^ = .09. T2 identification performance showed the characteristic pattern of lag-1 sparing, a subsequent decrease of accuracy for lags 2 and 3 (i.e., the attentional blink), and a recovery of performance for lag 7, *F*(3, 51) = 12.1, *p* < .0005, partial *η*^2^ = .42. Treatment and lag did not interact (*p* = .051). We found it remarkable that the treatment effects for lag 1 were of a similar magnitude as those on T1 accuracy. Indeed, although there was no overall effect of treatment, analysis of individual lags yielded a reliable effect of treatment for lag 1, *F*(2, 34) = 5.8, *p* = .007, partial *η*^2^ = .25, but not for the other three lags (all *p*s > .44). Pairwise comparisons for lag 1 revealed that accuracy in the clonidine (76.6 %; *t*_17_ = 3.1, *p* = .007) and scopolamine (79.1 %; *t*_17_ = 2.9, *p* = .009) conditions was lower than in the placebo condition (87.7 %), indicating that the treatment effects for T1 extended into lag 1 but not further. There was no reliable difference in accuracy between the clonidine and scopolamine conditions (*p* = .51).

Figure 3 (right panel) shows the results of a similar T2 analysis but constrained to T1-correct trials, as is common in attentional blink research. This analysis yielded similar statistical results. Treatment did not reliably influence T2 identification accuracy, *F* < 1. There was a main effect of lag, *F*(3, 51) = 18.6, *p* < .0005, partial *η*^2^ = .52, but no interaction between treatment and lag (*p* = .33). Again, separate analyses of the four lags yielded a reliable effect of treatment for lag 1 only, *F*(2, 34) = 3.3, *p* = .05, partial *η*^2^ = .16. Pairwise comparisons for lag 1 revealed that T2 accuracy was lower in the clonidine (92.3 %, *t*_17_ = 2.3, *p* = .04) and scopolamine (*t*_17_ = 2.1, *p* = .05) conditions than in the placebo condition (97.5 %). There was no reliable difference in accuracy between the clonidine and scopolamine conditions (*p* = .40).

### Electrophysiological data

**Fig. 4:**
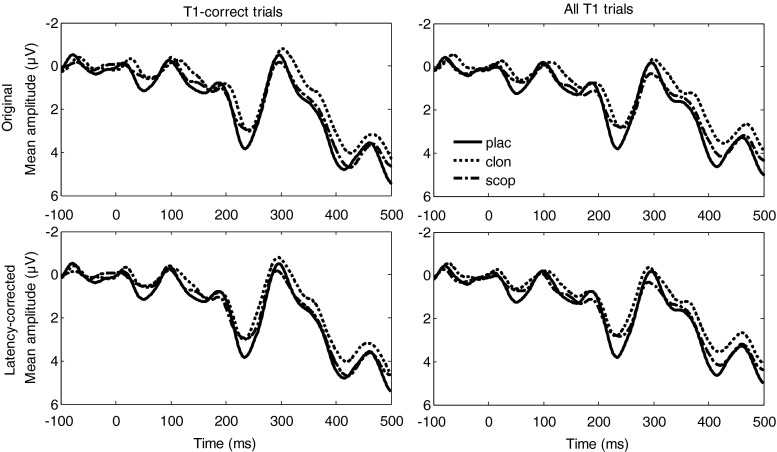
T1-locked grand average ERP waveforms for electrode Cz, plotted separately for each treatment. The *horizontal black bars* indicate time intervals where clonidine differed from placebo (see Methods and materials). *Upper panels*: original waveforms; *lower panels*: latency-corrected waveforms (see Results)

Because clonidine and scopolamine only reliably affected T1 accuracy, we next examined the effects of drugs on T1-evoked ERPs, separately for T1-correct trials (Fig. 4a) and for all trials, regardless of T1 accuracy (Fig. 4b). Importantly, as is evident in Fig. 4, the drug-related waveforms lagged behind the placebo-related waveform, which poses a problem for our sliding-window approach, because amplitude differences may in fact reflect latency differences. To deal with this issue, we first determined the size of the lag for each drug. To do so, we performed cross-correlations between the grand average waveforms for placebo and clonidine and for placebo and scopolamine (cf. Śmigasiewicz et al. 2014). We then shifted, sample by sample (in ~2-ms steps), the drug-related waveform relative to the placebo-related waveform and computed the cross-correlation across the time interval [0, 500 ms]. The maximum correlations were obtained by shifting the clonidine waveform 4 samples (~8 ms) to the left and by shifting the scopolamine waveform 2 samples (~4 ms) to the left. To correct for these lags, we next shifted the clonidine- and scopolamine-related waveforms of each subject by the corresponding duration. Grand average latency-corrected waveforms are shown in Fig. 4c, d. All ERP analyses reported below were based on these latency-corrected waveforms.

First, we examined the ERP waveforms for T1-correct trials. As can be seen in Fig. 4c, the sliding-window approach led to the identification of one significant time interval for electrode Cz: 217–273 ms. In this P2 window, clonidine was associated with a smaller mean amplitude (2.1 μV) than placebo (3.0 μV), *t*_17_ = 2.9, *p* = .01. Scopolamine (2.5 μV) did not differ from placebo, *t*_17_ = 1.1, *p* = .28.

Second, we examined the ERP waveforms based on all trials (i.e., regardless of T1 accuracy). The motivation for this analysis was that we were interested in the neural signatures associated with the drug-related impairment in T1 performance. Because several subjects made too few T1 errors to compute reliable error-specific ERP waveforms, we examined ERP waveforms based on all trials as neural signatures most representative of overall T1 performance. Here, the sliding-window approach led to the identification of two significant time intervals for electrode Cz (Fig. 4d): 217–273 ms, corresponding to the P2 component, and 373–430 ms, corresponding to the P3. In the P2 interval, clonidine was again associated with a smaller mean amplitude (2.1 μV) than placebo (2.9 μV), *t*_17_ = 3.0, *p* = .009, replicating the analysis based on T1-correct trials. In the P3 interval, clonidine was also associated with a smaller mean amplitude (2.9 μV) than placebo (3.8 μV), *t*_17_ = 2.9, *p* = .01. Both the P2 effect and the P3 effect were also significant for electrode CPz, suggesting a centroparietal locus of these effects. Examination of lag-specific T1-evoked ERPs indicated that the P2 and P3 effects were also present for longer lags, excluding the possibility that these effects constituted treatment effects on T2-related potentials that confounded the T1-evoked waveforms. Amplitudes for scopolamine generally lay in between clonidine and placebo (P2 interval 2.4 μV, P3 interval 3.5 μV) but did not differ reliably from placebo, both *p*s > .15.

Thus, relative to placebo, clonidine attenuated the amplitudes of the P2 and P3 components evoked by T1, although the latter effect was only reliable in the ERP analysis including correct and incorrect trials. Given that missed targets are typically associated with decreased P3 amplitudes (e.g., Rolke et al. 2001; dell’ Acqua et al. 2003), the effect of clonidine on P3 amplitude must have been driven at least in part by the increased number of included T1-incorrect trials.

## Discussion

In the present research, we investigated the effects of clonidine and scopolamine on multiple target detection in an RSVP context. In line with Nieuwenhuis et al. (2007), we found no effect of treatment on the attentional blink for T2. In contrast, we found that both clonidine and scopolamine impaired T1 accuracy. For clonidine, this effect was accompanied by a significant reduction in T1-evoked P2 and P3 amplitude.

The current study replicated Nieuwenhuis et al. (2007) with two design improvements: a crossover design instead of a between-subject design and a higher dose of clonidine, although, after a clinically relevant drop in blood pressure in one participant, the final seven participants were given the clonidine dose that was used by Nieuwenhuis et al. (2007). Like Nieuwenhuis et al. (2007), we found no effect of clonidine on the attentional blink, which poses a challenge for the attentional blink theory of Nieuwenhuis et al. (2005b) under the assumption that clonidine affects the phasic LC response. However, the evidence that addresses this assumption is limited. As in the present study, clonidine has been found to attenuate the amplitude of the P3 (Joseph and Sitaram 1989), which has been proposed to reflect phasic LC activity (Nieuwenhuis et al. 2005a). Furthermore, the suppressive effect of clonidine on tonic LC activity (e.g., Abercrombie and Jacobs 1987; Adams and Foote 1988), along with the reported interaction between tonic and phasic activity of the LC (Aston-Jones and Cohen 2005), suggests that clonidine may also affect sensory-evoked LC responses. However, the only study that we are aware of that directly examined this issue found mixed results: Adams and Foote (1988) found that during the onset of clonidine-induced suppressed LC firing, LC responses to sensory (footshock) stimuli were relatively preserved, although later during the experiment, the reliability of sensory-evoked LC responses was greatly reduced. Furthermore, in another experiment conducted as part of this study, we failed to find evidence that clonidine modulated the phasic alerting response to a task-irrelevant, auditory (“accessory”) stimulus, despite it having an effect on general alertness (Brown et al. 2015a). Thus, we may not have found an effect of clonidine on the attentional blink because it is possible that phasic LC responses to RSVP targets were preserved.

Alternatively, if clonidine affects the phasic LC response, as the observed reduction in T1-evoked P3 amplitude might indicate, the theory of Nieuwenhuis et al. (2005b) could be incorrect. For example, the size and the duration of the LC refractory period, which is purportedly mirrored in the attentional blink, may not be proportional to the size of the phasic LC response, as the theory suggests, or noradrenaline may not be involved in the attentional blink at all. At first sight, the study by de Martino et al. (2008) suggests a role of the noradrenergic system in the attentional blink: These authors found that administration of the β-adrenoceptor antagonist propranolol decreased T2 accuracy, while administration of the noradrenergic reuptake inhibitor reboxetine increased accuracy for emotional T2 stimuli. However, the authors found no interactions between lag and treatment, and in one of their experiments, propranolol impaired T1 accuracy as well. Taken together, these findings suggest that propranolol and reboxetine do not specifically modulate the attentional blink but target detection under RSVP conditions in general. More convincing evidence for noradrenergic modulation of the attentional blink was provided by Jepma et al. (2011), who studied patients with dopamine-β-hydroxylase deficiency, a rare genetic syndrome characterized by the complete lack of noradrenaline. They found that these patients had a larger attentional blink than healthy controls and that this impairment was restored by treatment with a synthetic precursor for noradrenaline. Although these findings pose a challenge for the theory of Nieuwenhuis et al. (2005a), which explains the attentional blink as a by-product of phasic noradrenaline release in the LC, they are generally consistent with a role for noradrenaline in the attentional blink. Recent studies have also reported evidence that decreased levels of dopamine in the striatum are associated with a larger attentional blink (Colzato et al. 2011; Colzato et al. 2008; Slagter et al. 2012). Other neurotransmitters may hence also play a role in the attentional blink.

In our study, clonidine had a clear detrimental effect on T1 identification accuracy. Presumably, clonidine impaired performance by reducing general alertness (e.g., Brown et al. 2015a; Coull et al. 1997; Coull 2001; Smith and Nutt 1996), a possibility that is supported by the negative effects of clonidine on simple reaction time, and the fact that scopolamine, which also increased simple reaction time, similarly reduced T1 identification accuracy. Nieuwenhuis et al. (2007) did not find a significant effect of clonidine on T1 accuracy. However, in their study, all participants took the lower clonidine dose, and their effect was in the same direction and of a similar magnitude (5 %) as the effect we observed here (6 %). To further understand the effect of clonidine on T1 accuracy, we examined T1-related ERP waveforms (Kenemans and Kähkönen 2011).

Clonidine attenuated the amplitude of the T1-evoked P2 and P3 components. The functional significance of the P2 is relatively ill-defined, but it has been related to some aspect of stimulus classification (reviewed in Key et al. 2005). The opposite seems to apply for the P3: Since its discovery in 1965, a number of theories have been proposed to account for its functional significance. In the context of the current paper, the work by Nieuwenhuis et al. (2005a) is particularly relevant, as these authors conceptualized the P3 as reflecting phasic noradrenergic activity and the concomitant increase in neural gain. As noted above, the clonidine-related decrease in P3 amplitude is consistent with several previous studies (Nieuwenhuis et al. 2005a; Joseph and Sitaram 1989). In contrast, previous studies have reported no effect of clonidine on the amplitude of the P2 (Abuljawad et al. 2001; Turetsky and Fein 2002). We propose that the effects of clonidine on T1-evoked P2 and P3 amplitudes and corresponding behaviors were mediated by a general decrease in alertness.

If clonidine reduced general alertness, why is that not manifested in reduced overall T2 accuracy? Relatedly, why did clonidine reduce lag-1 sparing? We propose that the perception of T1 caused a phasic alerting response that temporarily compensated for the drug-induced decrease in tonic alertness. As we have shown in other work, drug-related reductions in alertness yield room for compensatory accessory stimulus-induced performance improvements (Brown et al. 2015a). In a similar vein, Smith and Nutt (1996) found that arousal evoked by white noise can reduce the frequency of attentional lapses induced by clonidine intake. Furthermore, we propose that this phasic alerting response takes some time to unfold. This is suggested by our finding that the drug-related impairments in T1 accuracy extended to T2 accuracy if T2 was presented immediately after T1 (i.e., at lag 1). Only after that, from lag 2 onward, did accuracy return to placebo levels.

The scopolamine findings show a remarkable similarity to the clonidine findings. Like clonidine, scopolamine reduced T1 accuracy without having a clear effect on T2 accuracy. The reduction in T1 accuracy is generally consistent with a number of studies that have reported scopolamine-induced attentional impairments, as indicated by impaired performance in sustained attention tasks (Hasselmo and Sarter 2011). Scopolamine also led to reduced amplitudes of the P2 and P3 relative to placebo, although these reductions were not statistically significant.

It is possible that the effects of clonidine and scopolamine on behavior and ERP waveforms, though similar, were achieved via largely independent neural pathways that both affect general alertness. However, we believe that it is more plausible that the similar effects of these two drugs in the current study and another recent study in our lab (Brown et al. 2015a) reflect interactions between the two neuromodulator systems involved (Briand et al. 2007). On one hand, acetylcholine has been demonstrated to activate LC neurons in rats and co-administration of scopolamine reduces this effect (Egan and North 1985; Adams and Foote 1988). Egan and North proposed that scopolamine antagonizes muscarinic receptors in the LC, leading to reduced noradrenergic baseline activation. On that assumption, both clonidine and scopolamine may have reduced noradrenergic baseline activity, leading to a similar pattern of results for both treatments. On the other hand, there is solid evidence that clonidine inhibits cortical ACh release (Acquas et al. 1998), probably via α_2_ receptors in the basal forebrain (cf. Dringenberg and Vanderwolf 1998). This suggests that both clonidine and scopolamine may have reduced basal forebrain activity and consequent release of acetylcholine, thus leading to a similar pattern of results. The current data underline the importance of studying interactions between the noradrenergic and cholinergic neuromodulator systems in regulating temporal fluctuations in attention.

Some limitations of this study are worth mentioning. First, a general disadvantage of a within-subject design is that it is sensitive to practice effects. In our experiment, participants also became better at the task over the course of the three sessions. T2 accuracy increased with session, but this effect was not limited to the attentional blink; it occurred for all lags (cf. Slagter et al. 2007). This general practice effect likely contributed to increased error variance—T2 accuracy differences between sessions that were not due to treatment—decreasing the power to detect an existing effect. However, treatment was counterbalanced across sessions, and we found no effect of treatment order, suggesting that practice did not interact with treatment. Practice effects thus cannot easily account for the null effect of treatment on attentional blink magnitude and, more generally, T2 performance. A second potential limitation of our study is that the majority (15/18) of the participants were women and that the menstrual cycle was not taken into account and use of contraceptives was not registered. We are not aware of studies that have reported systematic performance differences in RSVP tasks as a function of sex, menstrual cycle, or use of contraceptives (but see Holländer et al. 2005), but we cannot exclude the possibility of such effects in our study.
